# Impact of Weight Loss on Plasma Leptin and Adiponectin in Overweight-to-Obese Post Menopausal Breast Cancer Survivors

**DOI:** 10.3390/nu7075156

**Published:** 2015-06-26

**Authors:** Henry J. Thompson, Scot M. Sedlacek, Pamela Wolfe, Devchand Paul, Susan G. Lakoski, Mary C. Playdon, John N. McGinley, Shawna B. Matthews

**Affiliations:** 1 Cancer Prevention Laboratory, Colorado State University, Fort Collins, CO 80523-1173, USA; E-Mails: john.mcginley@colostate.edu (J.N.M.); scomer@rams.colostate.edu (S.B.M.); 2 Rocky Mountain Cancer Centers, Denver, CO 80220, USA; E-Mails: scot.sedlacek@usoncology.com (S.M.S.); devchand.paul@usoncology.com (D.P.); 3 Colorado Biostatistics Consortium, University of Colorado, Denver, CO 80045, USA; E-Mail: pamela.wolfe@ucdenver.edu; 4 Department of Internal Medicine, University of Vermont, Burlington, VT 05405, USA; E-Mail: susan.lakoski@med.uvm.edu; 5 Department of Chronic Disease Epidemiology, Yale University, New Haven, CT 06520, USA; E-Mail: mary.playdon@yale.edu

**Keywords:** adiponectin, body composition, breast cancer survivors, dietary pattern, leptin, weight loss

## Abstract

Women who are obese at the time of breast cancer diagnosis have higher overall mortality than normal weight women and some evidence implicates adiponectin and leptin as contributing to prognostic disadvantage. While intentional weight loss is thought to improve prognosis, its impact on these adipokines is unclear. This study compared the pattern of change in plasma leptin and adiponectin in overweight-to-obese post-menopausal breast cancer survivors during weight loss. Given the controversies about what dietary pattern is most appropriate for breast cancer control and regulation of adipokine metabolism, the effect of a low fat *versus* a low carbohydrate pattern was evaluated using a non-randomized, controlled study design. Anthropometric data and fasted plasma were obtained monthly during the six-month weight loss intervention. While leptin was associated with fat mass, adiponectin was not, and the lack of correlation between leptin and adiponectin concentrations throughout weight loss implies independent mechanisms of regulation. The temporal pattern of change in leptin but not adiponectin was affected by magnitude of weight loss. Dietary pattern was without effect on either adipokine. Mechanisms not directly related to dietary pattern, weight loss, or fat mass appear to play dominant roles in the regulation of circulating levels of these adipokines.

## 1. Introduction

Prognosis for long-term survival following treatment for breast cancer is poorer in overweight or obese women with either pre- or postmenopausal breast cancer [1]. The prognostic disadvantage is accounted for by a higher risk of recurrence with subsequent metastatic progression and by the occurrence of cardiovascular disease and type-2 diabetes, which are common co-morbidities of breast cancer survivors [2]. Among women who were obese at the time of diagnosis as compared to normal weight women, a 33% higher risk for overall mortality has been reported in a meta-analysis of 43 studies [3]. A number of mechanisms have been proposed to mediate prognostic disadvantage, including those involving two adipokines, adiponectin and leptin [4,5,6,7,8]. However, the clinical significance of these findings remains controversial; many studies were conducted using cross-sectional designs that failed to establish temporal relationships for causality and results have been inconsistent.

The primary intervention used to treat obesity-associated diseases is weight loss via lifestyle modifications involving energy intake and expenditure [9]. In post-menopausal women, limiting caloric intake rather than increasing energy expenditure via physical activity is the key to achieving weight loss [10]. Of the dietary patterns used for weight loss, those that have received the most attention, low carbohydrate or low fat, are the same patterns that are at the center of a controversy about dietary approaches for breast cancer prevention and control [11,12,13,14,15,16,17,18,19,20,21,22,23,24]. In both weight control and cancer control, many studies have focused on low-fat diets; however, proponents of low carbohydrate diets report that high carbohydrate intake results in higher plasma insulin levels and promotes lipogenesis [25]; hence the popularity of low-carbohydrate diets for weight loss among the general population. Clearly, the same characteristics associated with low fat weight loss diets, *i.e.*, higher insulin and promotion of lipogenesis, would be considered to be a prognostic disadvantage in the breast cancer survivor population [6,26].

This paper focuses on the effects of intentional weight loss achieved using two different dietary patterns on circulating concentrations of leptin and adiponectin. These adipokines are the most abundant proteins synthesized and secreted by adipocytes. Adiponectin and leptin have been reported to play roles in obesity as well as obesity-associated diseases such as cardiovascular disease, type-2 diabetes, and cancer, including breast cancer [27,28,29,30,31]. Adiponectin is 1000 times more abundant in plasma than is leptin. Leptin has been reported to be positively associated with fat mass; whereas, plasma adiponectin has been reported to increase as fat mass decreases. The mechanisms that regulate synthesis and secretion of these adipokines *in vivo* are not well understood. In general, both proteins appear to act via binding to their cognate receptors. Adiponectin has been reported to be insulin sensitizing and to antagonize the effects of leptin on the carcinogenic process in the breast [32,33,34,35]. Leptin is associated with eating behavior and has been reported to promote breast carcinogenesis and tumor progression via effects on multiple regulatory nodes that drive mitogenesis while blocking apoptosis and enhancing angiogenesis [29,36,37].

This study, referred to as CHOICE, was designed to investigate how weight loss induced using two dietary patterns, low fat or low carbohydrate, affected biomarkers that have been reported to impact breast cancer recurrence and other common comorbidities of breast cancer survivors, *i.e.*, type 2 diabetes and cardiovascular disease. Herein we report on adiponectin and leptin. We hypothesized that adiponectin would increase and leptin would decrease in a manner directly proportional to weight loss and decrease in fat mass and a low carbohydrate dietary pattern would induce a more favorable response. Unlike most other studies of weight loss, anthropometric data were collected monthly so that changes in the rate of weight loss and body fat mass could be computed and provide insight into patterns of change in circulating concentrations of leptin and adiponectin and whether the dietary approach to weight loss had any impact on the outcomes observed.

We report that while leptin was strongly associated with fat mass at baseline, adiponectin was not, and the lack of correlation between changes in leptin and adiponectin implies independent mechanisms of regulation. The temporal pattern of change in leptin and adiponectin varied markedly and was not directly related to change in fat mass. Dietary pattern was without effect on plasma concentrations of either adipokine. Since adiponectin and leptin have been reported to have opposing activities on breast cancer progression, we also assessed whether their ratio might provide an index of intervention effectiveness but found that the variability of the ratio makes it problematic in this regard.

## 2. Experimental Section

### 2.1. Study Design

This study, referred to as CHOICE, compared two weight loss interventions that differed only in the dietary pattern that was investigated, *i.e.*, low carbohydrate *versus* low fat with dietary protein content equivalent in both interventions. The details of the CHOICE research protocol have been published [38]. Briefly, participants were followed for 6 months. Demographic and anthropometric data were collected at baseline and we continued to measure weight, waist and hip circumference, body mass index, and body composition via Bod Pod^®^ (Life Measurement, Inc., Concord, CA, USA) at 4-week intervals thereafter up to six months. The clinical protocol was approved by the Colorado State University Institutional Review Board for the Protection of Human Subjects. Written consent was obtained before enrolling participants.

### 2.2. Participants

Women recruited for participation were from a single oncology practice and were at least 4 months post chemotherapy, radiation and surgical treatment for breast cancer and were considered clinically free of cancer. Accrual occurred from 2008 to 2012. Participants were referred by their medical oncologist and had a body mass index in the overweight or obese class I range (BMI 25.0–34.9Kg/m^2^). Eligibility criteria have been reported elsewhere [38]. A total 249 participants were assigned to the study (Supplementary Figure S1). Clinical characteristics and demographic data across groups at baseline are shown in Supplementary Table S1. Participants were predominately non-Hispanic whites (89%) with a mean age of 54.9 ± 9.2 years, a mean BMI of 29.0 ± 2.6 kg/m^2^ and an average of 43 ± 5% body fat. There were no differences among study arms in clinical or demographic characteristics, including disease stage or treatment regime. During the course of the study, dropout rate was similar in the low carbohydrate (15%) and the low fat (18%) study arms, although it was higher in the non-intervention control (26%); the differences were not statistically significant (*p* = 0.22). Demographics for those lost to follow up were not different from those who completed the study, with the exception of time since end of treatment (*p* = 0.01). Overall compliance with the menu and recipe defined dietary patterns was determined using the daily food logs kept by each participant and that were reviewed at each monthly clinic visit. The mean compliance for the 6-month intervention was 73% (monthly range: 65%–81%) and was not different by intervention arm (*p* = 0.80).

### 2.3. Adverse Events

One participant was treated for a pulmonary embolism, one participant was treated at an emergency room for stomach pain, two participants were treated for falls, one of which resulted in a hairline hip fracture, and one individual experienced an allergic reaction to an antibiotic. The adverse events were not attributed to the weight loss intervention.

### 2.4. Non Intervention Control

Individuals not interested in joining the weight loss arms of the study but who wished to participate were assigned to the non-intervention control group.

### 2.5. Intervention

Two interventions were developed and were comprised of a structured diet and physical activity program with daily recording of body weight and activity. Each program was designed to create a weekly negative energy balance equivalent to 3500 kcal, after adjustments for metabolic adaptations that occur during extended periods of weight loss; however, participants were not limited in the amount of weight they lost and individuals who lost more than the target weight were considered compliant if they followed their prescribed dietary pattern. The diet plan for each intervention arm was comprised of a 42-day cycle of menus and recipes that were designed for five calorie levels in each intervention arm (Supplementary Table S2). The meal plans included interchangeable meal options (home-prepared recipes and meal instructions; eating out and convenience meal options), educational material and a program incorporating weight loss strategies based on a systematic review of the literature. The intervention was designed as a feeding study but was conducted in free living individuals, where strict dietary structure is presented in a format that also offers enough flexibility to be adopted into daily living and by the families and social support networks of participants.

### 2.6. Laboratory Analyses

For measurement of leptin and adiponectin, EDTA plasma samples were assayed with solid phase quantitative sandwich ELISA (R&D systems, Minneapolis, MN, USA) according to standard protocol. All assays were carried out blinded to intervention arm assignment. Leptin and adiponectin interassay coefficients of variation ranged from 4.9% to 8.1% and from 10.0% to 11.3%, respectively.

### 2.7. Statistical Methods

Analysis was restricted to the subjects who completed the study, as the effects of 6 months of weight loss were of primary interest in these analyses. Correlations at baseline and 6 months were estimated using spearman’s rank statistic. Six-month changes in weight and body composition for each diet group *vs.* control were evaluated in an ANCOVA model. All other inference was done on longitudinal data using a maximum likelihood model for repeated measures. The correlation structure was assumed to be autoregressive, except in the analysis of leptin and adiponectin, where compound symmetry was used due to lack of convergence. Because diet group was not assigned at random, baseline body mass index, resting metabolic rate and elapsed time since the end of cancer treatment were added to all regressions; the time varying covariate steps was also included. Differences across visit and between diet group were estimated using linear contrasts. For the question of whether fat loss or time on study was more important, change in fat mass was added to the regression model. All analyses were done in SAS 9.3, SAS Inc., Cary, NC, USA. GraphPad Prism 5.0 (GraphPad Software, Inc., La Jolla, CA, USA) was used to visualize the data.

## 3. Results

### 3.1. Anthropometric Determinates

Body weight and fat mass decreased in each of the six-month weight loss interventions (Table 1). Body weight and fat mass were lower at the end of the six-month intervention in each intervention arm compared to the control group (*p* < 0.001). Percent body fat was lower in the low fat than the low carbohydrate intervention arm at all time points; however, the slope of the regression lines describing the rate of change in percent body fat over time did not differ between intervention arms.

**Table 1:** Anthropometric measurements over time ^1^.

Variable	Group	Baseline	Month 1	Month 2	Month 3	Month 4	Month 5	Month 6
**Body Weight (kg)**	CTRL	53, 79.7 ± 9.3 (11.7)						53, 79.4 ± 10.1 (12.7)
	LC	65, 79.8 ± 8.7 (10.9)	65, 76.0 ± 8.4 (11.1)	65, 74.0 ± 8.4 (11.4)	64, 72.3 ± 8.4 (11.7)	62, 71.2 ± 8.7 (12.2)	61, 70.2 ± 8.9 (12.6)	65, 69.4 ± 9.0 (13.0)
	LF	73, 77.6 ± 7.7 (9.9)	73, 74.2 ± 7.4 (10.0)	71, 72.3 ± 7.5 (10.3)	69, 71.1 ± 7.5 (10.5)	69, 70.0 ± 7.4 (10.6)	67, 69.0 ± 7.6 (11.0)	73, 68.3 ± 7.5 (11.0)
**Fat Mass (kg)**	CTRL	53, 34.9 ± 7.3 (20.9)						53, 34.9 ± 8.2 (23.4)
	LC	65, 35.0 ± 6.1 (17.4)	65, 31.9 ± 5.7 (18.0)	65, 30.2 ± 5.6 (18.7)	64, 28.5 ± 5.8 (20.5)	62, 27.4 ± 6.2 (22.5)	61, 26.1 ± 6.4 (24.5)	65, 25.4 ± 6.6 (26.1)
	LF	73, 33.0 ± 5.8 (17.6)	73, 29.8 ± 5.7 (19.2)	71, 28.1 ± 5.6 (19.8)	69, 26.7 ± 5.8 (21.6)	69, 25.7 ± 5.6 (21.8)	67, 24.8 ± 5.9 (23.9)	73, 24.1 ± 5.7 (23.7)
**Waist (cm)**	CTRL	53, 94.9 ± 8.3 (8.7)						53, 94.8 ± 8.8 (9.3)
	LC	65, 94.3 ± 6.9 (7.4)	65, 91.6 ± 7.5 (8.2)	65, 89.8 ± 7.6 (8.4)	64, 87.8 ± 7.6 (8.6)	62, 87.1 ± 7.7 (8.9)	61, 86.0 ± 7.7 (8.9)	65, 85.0 ± 7.5 (8.8)
	LF	73, 91.6 ± 7.2 (7.9)	73, 89.0 ± 6.8 (7.6)	71, 86.7 ± 7.0 (8.1)	69, 85.6 ± 7.8 (9.1)	69, 84.4 ± 7.0 (8.3)	67, 83.5 ± 7.3 (8.7)	73, 83.1 ± 7.4 (8.9)
**Hip (cm)**	CTRL	53, 110.5 ± 7.4 (6.7)						53, 111.0 ± 9.2 (8.3)
	LC	65, 112.0 ± 7.2 (6.4)	65, 109.3 ± 6.9 (6.3)	65, 107.4 ± 6.9 (6.5)	64, 106.1 ± 7.3 (6.9)	62, 104.7 ± 7.3 (7.0)	61, 103.9 ± 8.2 (7.9)	65, 103.2 ± 7.3 (7.1)
	LF	73, 110.7 ± 5.8 (5.2)	73, 107.6 ± 5.8 (5.3)	71, 105.9 ± 6.2 (5.8)	69, 105.1 ± 5.4 (5.1)	69, 103.7 ± 5.3 (5.1)	67, 102.8 ± 6.1 (6.0)	73, 102.2 ± 5.6 (5.5)
**WHR**	CTRL	53, 0.9 ± 0.1 (8.2)						53, 0.9 ± 0.1 (9.1)
	LC	65, 0.8 ± 0.1 (7.9)	65, 0.8 ± 0.1 (7.9)	65, 0.8 ± 0.1 (9.0)	64, 0.8 ± 0.1 (8.1)	62, 0.8 ± 0.1 (7.6)	61, 0.8 ± 0.1 (8.5)	65, 0.8 ± 0.1 (7.5)
	LF	73, 0.8 ± 0.1 (6.7)	73, 0.8 ± 0.1 (6.4)	71, 0.8 ± 0.1 (6.7)	69, 0.8 ± 0.1 (7.5)	69, 0.8 ± 0.1 (6.7)	67, 0.8 ± 0.1 (6.7)	73, 0.8 ± 0.1 (6.8)

^1^ Values are *n*, means ± SD (CV); CTRL, control; LC, low carbohydrate; LF, low fat; and WHR, waist hip ratio.

There are two primary sites of lipid storage, central (abdominal) and peripheral (subcutaneous), and storage of fat in these depots is estimated by measuring waist and hip circumference, respectively. Both measures decreased progressively with fat loss, and there was only a slight but statistically significant change in the WH ratio (*p* = 0.04), indicating that waist circumference decreased slightly more than hip circumference (Table 1). Total fat lost, change in percent body fat, and change in the WH ratio were unaffected by the dietary pattern used to induce weight loss.

### 3.2. Fat Mass and Plasma Concentrations of Leptin and Adiponectin

**Figure 1:**
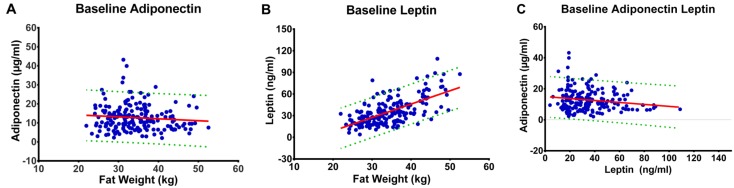
Regression Analyses of Baseline data: (**a**) Regression of plasma adiponectin on fat mass (kg) with 95% confidence intervals; r^2^ = 0.008, *p* = 0.210. (**b**) Regression of plasma leptin on fat mass (kg) with 95% confidence intervals; r^2^ = 0.41, *p* < 0.001. (**c**) Regression of plasma adiponectin on plasma leptin with 95% confidence intervals; r^2^ = 0.025, *p* = 0.028.

Because leptin and adiponectin are synthesized primarily by adipocytes and secreted into blood, we next used baseline data to determine the pre-intervention relationship between fat mass and plasma concentrations of leptin and adiponectin (Figure 1). Fat mass explained 35% of the variability in leptin (r^2^ = 0.41); whereas, fat mass explained only 1% of the variability in plasma adiponectin (r^2^ = 0.008). Given these findings, it was not surprising that regression analysis showed no association between plasma leptin and adiponectin (r^2^ = 0.025). The same analyses were done at end of study, after an overall decrease in fat mass of 27.3%, and very similar r^2^ values were found (Supplementary Figure S2).

### 3.3. Pattern of Change

**Figure 2:**
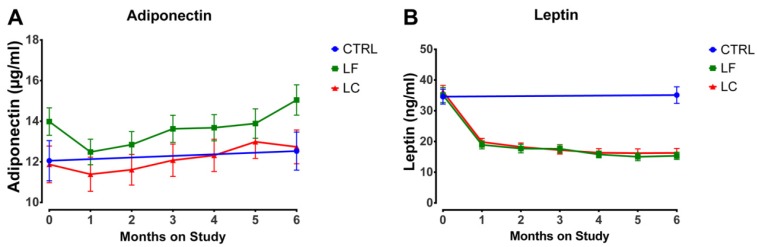
Plasma adipokine concentrations duration weight loss. Groups are non intervention control, CTRL; low fat dietary pattern weight loss intervention arm, LF; and low carbohydrate dietary pattern weight loss intervention arm, LC. Values are means ± SD. (**a**) Plasma adiponectin (μg/mL) as a function of months on weight loss intervention. The decrease in plasma adiponectin observed after the first month of weight loss (6%, *p* < 0.001 relative to baseline) was observed in both intervention arms. Thereafter, plasma adiponectin increased (*p* = 0.004), but was not different from the control in either intervention group by end of study. Although adiponectin levels were greater in the low fat group, the rate of increase in adiponectin from intervention months 1 to 6 in the two diet groups were approximately the same (*p* = 0.85); the increase in the last month of the intervention was somewhat greater in the low fat group than the low carbohydrate group (*p* = 0.03). (**b**) Plasma leptin (ng/mL) as a function of months on weight loss intervention. There was a large decrease in leptin over the first month of weight loss (81% of the six-month decrease) with relatively small decreases thereafter. This pattern of change in leptin was observed in both intervention arms and the decrease in leptin was unaffected by dietary pattern.

We examined the temporal pattern of change in leptin and adiponectin (Figure 2 and Table 2). Given the strong correlation between fat mass and plasma leptin at baseline and end of study, we expected that leptin would decrease as fat mass decreased, but it did not. Rather, there was a large decrease in leptin over the first month of weight loss (81% of the six-month decrease) with relatively small decreases thereafter, while fat mass decreased only 34% of the six-month total reduction during the first month of the intervention. This pattern of change in leptin was observed in both intervention arms and the decrease in leptin was unaffected by dietary pattern.

Despite the very low correlation with fat mass at baseline, we expected plasma levels of adiponectin to increase throughout weight loss based on published reports [39,40]. Thus the marked decrease in plasma adiponectin observed after the first month of weight loss (6%, *p* < 0.001 relative to baseline) was unexpected and was observed in both intervention arms. Thereafter, plasma adiponectin increased (*p* = 0.004), but was not different from the control in either intervention group by end of study. Although adiponectin levels were greater in the low fat group, the rate of increase in adiponectin from intervention months 1 to 6 in the two diet groups were approximately the same (*p* = 0.85); the increase in the last month of the intervention was somewhat greater in the low fat group than the low carbohydrate group (*p* = 0.03).

Because computation of the ratio of adiponectin to leptin has been suggested as an index for assessing net effects of these adipokines on diseases like breast cancer, the ratio was also evaluated. However, we found that the coefficient of variation (CV) for the ratio was extremely high (92%), whereas the CVs for leptin, adiponectin and fat mass were 51%, 53%, and 18%, respectively. Similar values were found at end of study. The high variability in the ratio suggests it would not be a sensitive marker for meaningfully assessing biological effects.

**Table 2:** Adiponectin and leptin with change relative to baseline ^1^*.*

Variable	Group		Baseline		Month 1		Month 2		Month 3		Month 4		Month 5		Month 6	
**Adiponectin (μg/mL)**	CTRL		53, 12.1 ± 7.2 (59.8)												52, 12.5 ± 6.8 (54.0)	
	LC		65, 11.9 ± 7.3 (61.3)		65, 11.4 ± 6.8 (59.6)		65, 11.6 ± 6.1 (52.6)		63, 12.1 ± 6.3 (52.3)		62, 12.3 ± 6.2 (50.7)		61, 13.0 ± 6.4 (49.4)		65, 12.7 ± 6.7 (52.9)	
	LF		72, 14.0 ± 5.7 (41.0)		73, 12.5 ± 5.4 (43.1)		71, 12.8 ± 5.4 (42.4)		69, 13.6 ± 5.6 (40.8)		69, 13.7 ± 5.4 (39.2)		66, 13.9 ± 5.9 (42.2)		72, 15.0 ± 6.3 (42.1)	
**Adiponectin (%) ∆ from baseline**	CTRL														52, 10.6 ± 38.7 (363)	
	LC				65, −3.4 ± 20.7 (−615)		65, 2.5 ± 23.2 (925)		63, 6.8 ± 25.7 (381)		62, 10.5 ± 32.3 (309)		61, 14.1 ± 32.7 (231)		65, 13.0 ± 25.7 (198)	
	LF				72, −7.8 ± 21.8(−279)		71, −6.0 ± 25.1 (−420)		69, 1.1 ± 23.3 (2093)		69, 2.9 ± 27.7 (961)		66, 2.7 ± 24.5 (918)		71, 12.1 ± 27.0 (223)	
**Leptin (ng/mL)**	CTRL		53, 34.6 ± 17.7 (51.2)												52, 35.1 ± 19.4 (55.3)	
	LC		65, 36.5 ± 16.2 (44.5)		65, 20.0 ± 9.7 (48.5)		65, 18.4 ± 10.5 (57.2)		63, 17.3 ± 10.4 (60.0)		62, 16.5 ± 10.6 (64.4)		61, 16.4 ± 11.1 (68.0)		65, 16.4 ± 11.8 (71.8)	
	LF		72, 35.1 ± 20.7 (59.1)		73, 18.9 ± 11.2 (59.0)		71, 17.7 ± 11.7 (66.4)		69, 17.6 ± 11.3 (63.9)		69, 15.8 ± 9.0 (57.2)		66, 15.0 ± 10.5 (69.7)		72, 15.3 ± 9.8 (64.0)	
**Leptin (%) ∆ from baseline**	CTRL														52, 6.5 ± 41.7 (643)	
	LC				65, −43 ± 20.1 (−47)		65, −46 ± 25.9 (−56)		63, −50 ± 23.5 (−47)		62, −53 ± 24.5 (−46)		61, −53 ± 26.2 (−49)		65, −52 ± 30.6 (−59)	
	LF				72, −44 ± 17.6 (−40)		71, −48 ± 20.5 (−42)		69, −48 ± 24.0 (−50)		69, −53 ± 18.6 (−35)		66, −54 ± 24.2 (−45)		71, −51 ± 31.9 (−62)	
**Ratio Adiponectin/eptin**	CTRL		53, 0.5 ± 0.4 (87.0)												52, 0.5 ± 0.4 (78.0)
	LC		65, 0.4 ± 0.5 (119)		65, 0.8 ± 1.0 (123)		65, 0.9 ± 1.0 (112)		63, 1.1 ± 1.3 (121)		62, 1.2 ± 1.3 (114)		61, 1.4 ± 2.5 (175)		65, 1.4 ± 2.1 (144)
	LF		72, 0.6 ± 0.4 (75.1)		73, 0.9 ± 0.6 (68.5)		71, 1.1 ± 0.9 (83.5)		69, 1.2 ± 1.0 (87.0)		69, 1.3 ± 1.1 (85.4)		66, 1.4 ± 1.4 (96.7)		72, 1.6 ± 1.7 (108)

^1^ Values are *n*, means ± SD (CV). CTRL, control; LC, low carbohydrate; LF, low fat.

### 3.4. Impact of Weight Loss Magnitude

**Figure 3:**
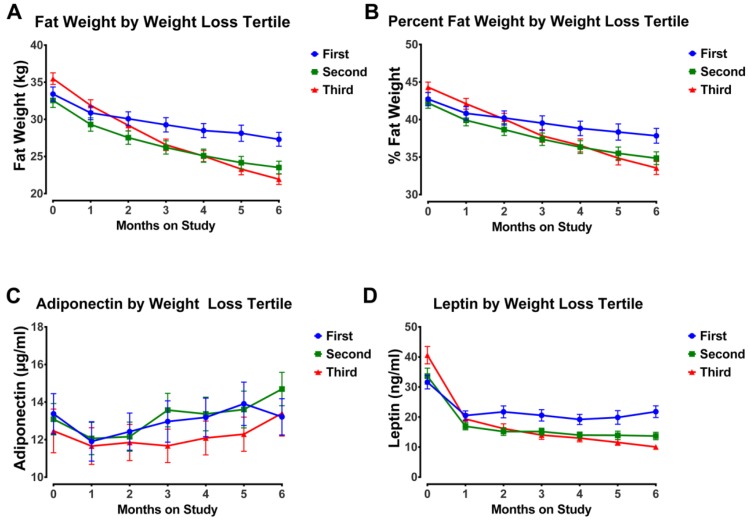
Body fat mass and plasma adipokines by weight loss tertile. The subset of individuals who lost at least 5% of initial body weight (92% of all individuals who completed the study) were divided into tertiles of weight loss, which corresponded to >5%, tertile 1; >10%, tertile 2; and >15%, tertile 3 of initial body weight. (**a**) Fat mass in kg over successive months of the weight loss intervention. (**b**) Body fat expressed as a (%) over successive months of the weight loss intervention. (**c**) Adiponectin (μg/mL) over successive months of the weight loss intervention. (**d**) Leptin (ng/mL) over successive months of the weight loss intervention. Values are means ± SEM. With weight loss >10%, leptin decreased progressively over time, and the decrease was significant with weight loss >15% (*p* = 0.007); whereas, there was no significant effect of weight loss magnitude on circulating levels of adiponectin.

The analyses performed to this point indicated that 35% of the variation in leptin and only 1% of the variation in plasma adiponectin could be accounted for by fat mass at baseline and that change in leptin was far more pronounced in the first month of the intervention, while loss of fat mass continued throughout. An alternative hypothesis to explain change in the plasma concentrations of these adipokines is that magnitude of energy restriction was exerting effects on circulating leptin and adiponectin independent of body composition. To evaluate this possibility, the subset of individuals who lost at least 5% of initial body weight (92% of all individuals who completed the study), a level that is considered clinically meaningful [2], were divided into tertiles of weight loss, which corresponded to >5%, >10%, and >15% of initial body weight. Plasma leptin and adiponectin concentrations were plotted over time by weight loss tertile (Figure 3). It is clear that with weight loss >10%, leptin decreased progressively over time, and the decrease was significant with weight loss >15% (*p* = 0.007); whereas, there was no significant effect of weight loss magnitude on circulating levels of adiponectin.

### 3.5. Clinical Relevance of Changes in Plasma Adipokines

**Figure 4:**
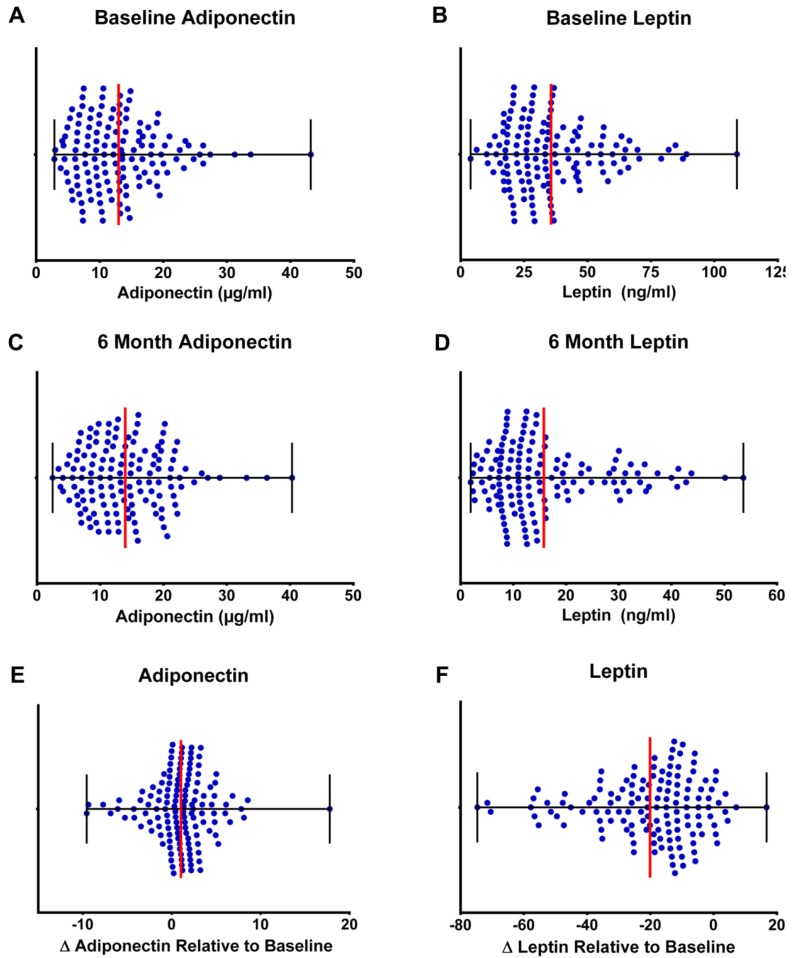
Distribution of plasma adipokines at baseline and end of study. (**a**) Scatterplot showing the mean and range for plasma adiponectin and leptin at baseline. The plasma concentration of adiponectin that is observed in normal weight individuals is 7–5 µg/mL. Greater than 50% of participants in CHOICE were within this range at baseline. A concentration of 5 to 10 ng leptin per mL is typically observed in normal weight individuals. In this study, the mean leptin concentration at baseline was 36.0 ± 18.5 ng/mL. (**b**) Scatterplot showing the mean and range for plasma adiponectin and leptin at end of study. The plasma concentration of adiponectin that is observed in normal weight individuals is 7–15 µg/mL and 47% remained within this range by end of study and those percentages were not significantly affected by weight loss tertile or dietary pattern. Plasma leptin decreased to 15.8 ± 10.8 ng/mL by end of study; however, only in the highest tertile of weight loss did plasma levels reach the normal range of plasma concentrations (baseline 40.6 ±19.6 down to 10.0 ± 5.7 ng/mL). (**c**) Scatterplot showing the mean and range for the change (end of study-baseline) in plasma adiponectin (μg/mL) and leptin (ng/mL) at end of study relative to baseline. The distribution of change in plasma adiponectin indicates that 32% of the individuals in the intervention arms experienced a six-month decrease in adiponectin; whereas, the remainder increased somewhat. The distribution of change in plasma leptin indicates that 92% of participants in the weight loss intervention experienced a decrease in plasma leptin.

A concentration of 5 to 10 ng leptin per mL is typically observed in normal weight individuals [41]. In this study, the mean leptin concentration at baseline was 36.0 ± 18.5 ng/mL and in the intervention arms decreased to 15.8 ± 10.8 by end of study (Figure 4); however, only in the highest tertile of weight loss did plasma levels reach the normal range of plasma concentrations (baseline 40.6 ± 19.6 down to 10.0 ± 5.7 ng/mL). The distribution of change (baseline to end of study) in plasma leptin in the CHOICE cohort (Figure 4) indicates that 92% of participants in the weight loss intervention experienced a decrease in plasma leptin.

The plasma concentration of adiponectin that is observed in normal weight individuals is 7–15 µg/mL [42]. Greater than 50% of participants in CHOICE were within this range at baseline and 47% remained within this range by end of study and those percentages were not significantly affected by weight loss tertile or dietary pattern (Figure 4). Plasma adiponectin in the highest weight loss tertile was 12.7 ± 7.7 at baseline and 13.7 ± 8.0 at end of study. The distribution of change in plasma adiponectin indicates that 32% of the individuals in the intervention arms experienced a six-month decrease in adiponectin; whereas, the remainder increased somewhat (Figure 4).

## 4. Discussion

Reports on the relationship of circulating levels of adiponectin and leptin to incident post-menopausal breast cancer are contradictory [27,43,44,45,46,47,48], and the association of breast cancer recurrence and survival with circulating concentrations of these adipokines is equally mixed [4,5,6,7,8]. This study was not designed to directly answer questions about effects of intentional weight loss on recurrence or survival or whether adiponectin or leptin play a causal role in this regard. Rather, the goal was to define how leptin and adiponectin are affected by weight loss and dietary pattern as a first step in establishing whether those changes support the biological plausibility for these adipokines to play a causal role in weight loss mediated effects. Because of the monthly assessment of anthropometric endpoints and collection of plasma in CHOICE, our results sled light on how circulating levels of leptin and adiponectin change in relation to changes in body weight, body fat, and weight loss dietary pattern. The expectations that we had from the preclinical and clinical literature were: (1) that leptin would decrease and adiponectin would increase in a manner proportional to weight loss; (2) that there would be a strong relationship between plasma leptin and adiponectin and both fat mass and changes in fat mass; (3) that there would be a strong relationship between leptin and adiponectin as well as changes in leptin and adiponectin throughout the weight loss intervention; and (4) that changes in leptin and adiponectin would be influenced by dietary pattern. The extent to which these expectations were supported by our findings forms the basis for the remainder of the discussion.

### 4.1. Leptin

Leptin decreased during the six-month weight loss intervention (Figure 2) and the 52% reduction observed between baseline and end of study is consistent with other reports [39,40,49,50]. By assessing weight loss and plasma leptin concentrations monthly, it became clear that the changes in leptin were not directly proportional to the amount of weight loss or to loss of fat mass *per se*. Rather, most change in leptin occurred during the first month of the intervention with limited reductions thereafter (Figure 2). Given the dramatic decrease in leptin during this timeframe and that significant remodeling of adipose tissue is reported to accompany weight loss [51], the manner in which leptin synthesis and secretion are affected by tissue remodeling merits consideration. We also observed that weight loss greater than 15% resulted in significant additional decreases in plasma leptin between one and six months (Figure 3) but that dietary pattern was without effect (Figure 2). These findings are consistent with reports that energy restriction decreases leptin gene expression in white fat adipocytes and that magnitude of energy restriction plays a role [52]. The same dietary patterns that were used in CHOICE were also found to be without effect on leptin gene expression in the weight loss context [52]. Thus, despite a mixture of reports about the effects of dietary patterns on circulating concentrations of leptin [49,53,54,55,56,57,58,59,60,61,62,63], our findings are very clear-cut and also in agreement with another study in a population of breast cancer survivors in which effects of weight loss and dietary pattern have been reported [49]. Given that the manner in which leptin gene expression is regulated remains poorly understood [64], these findings indicate the need to look beyond linkages of leptin to fat mass or body weight *per se* in efforts to identify new approaches to modulating the secretion of leptin from adipose tissue. In terms of a potential causal role of leptin as a driver of breast carcinogenesis, preclinical data show that leptin regulates JAK2/STAT3 and inflammatory cytokine related signaling, which is altered by obesity and reregulated via weight loss [65,66]. However, two clinical reports fail to link circulating leptin with breast cancer recurrence [4,5], although another has shown leptin to be associated with distant recurrence and death even when statistical models were adjusted for BMI and body weight [6]. Plasma leptin data have to be interpreted with caution since leptin and the two main isoforms of its receptor have been reported to be expressed in 84% of breast cancers suggesting that cells within tumors can respond to leptin via autocrine as well as paracrine and endocrine pathways [67].

### 4.2. Adiponectin

If the focus of CHOICE was simply on assessing the plasma concentration of adiponectin at baseline and end of study, we would have concluded that there was no effect of weight loss on this adipokine, which is consistent with one report [49] but at odds with others [39,40]. However, the monthly collection of data provided clear evidence that after an initial fall in adiponectin, which was consistent with another report [42], there was a small but progressive increase observed over the remaining five months of the study. Moreover, our findings show that changes in adiponectin were not significantly linked to either loss in body weight or fat mass, or the magnitude of weight loss (Table 1 and Table 2). These findings fail to support commonly stated perceptions about the relationship of adiponectin to body mass, body composition or weight control. Similarly, and consistent with [49], CHOICE data indicate that neither the low carbohydrate or low fat dietary patterns had an effect on changes in plasma adiponectin concentration in the weight loss context, despite many reports indicating dietary composition would impact circulating concentrations of this adipokine [56,58,59,61,63,68,69,70,71]. Collectively, the CHOICE results support an earlier study that reported that weight loss using either a low fat or a low carbohydrate pattern had no effect on adiponectin gene expression in white adipose tissue [52].

Of particular interest to us was the dichotomous nature of the change in adiponectin that was observed in the CHOICE cohort, which differed markedly from the predominant decrease observed in plasma leptin (Figure 4). While the net effect of weight loss on plasma adiponectin was null, about 32% of the population experienced a net decrease; and the remainder a net increase. The factors that account for the differences in response in these two subpopulations are unclear, but could relate to polymorphisms in the adiponectin gene or to differences among individuals that determine whether synthesized adiponectin is secreted into the circulatory system or retained within the cell and degraded via lysosomal metabolism [42]. Nonetheless, the normal range for plasma adiponectin is 7–15 µg/mL and most of the CHOICE population remained within this range throughout the intervention.

Preclinical data provide a clear set of pathways that are impacted by adiponectin and that are linked to tumorigenesis, particularly tumor progression. Prominent among these are the activation of AMP-activated protein kinase, and deactivation of acetyl CoA carboxylase, effects that are considered to protective against cancer [72]. However, as with leptin, evidence linking adiponectin to recurrence or survival is mixed [4,5,6,7], and adiponectin and its receptors have been detected in breast cancer tissue indicating that autocrine as well as paracrine and endocrine pathways are operative [27]. Thus circulating adipokine data alone are unlikely to be sufficient to determine causal relationships. In line with this observation, it is worth noting that interpretation of plasma adiponectin data relative to health benefits is complex. Some reports indicate that vascular health and the predisposition to diabetes are negatively affected by adiponectin levels below 6 μg/mL, and that increased risk of all cause and cardiovascular mortality is observed above 12.2 μg/mL [73]. When these values are juxtaposed to the 15.5 μg/mL plasma concentration of adiponectin below which breast cancer survival has been reported to be adversely affected (above the median: 15.5 μg/mL, longer breast cancer survival, HR, 0.39; 95% CI, 0.15 to 0.95, [74]), it would seem premature to consider adiponectin of value in assessing prognosis for survival benefit, particularly in the weight loss setting.

### 4.3. Adiponectin to Leptin Ratio and Causal Mechanisms

There have been many reports suggesting the use of the ratio of adiponectin to leptin to infer cancer risk [30,31,40,75]. In support of that concept is our finding that neither plasma concentration of adiponectin or leptin nor change in adiponectin and leptin were statistically correlated indicating that each adipokine is providing independent information. Similarly, there are several recent reviews detailing candidate mechanisms by which leptin and adiponectin could be impacting various aspects in the development of cancer in an integrated manner [65,66,72]. However, across the CHOICE cohort, the coefficient of variation in the adiponectin to leptin ratio at any time point assessed was between 90% and 120%. Moreover, when we determined whether the ratio increased monotonically within an individual during progressive weight loss, it did so in less than 10% of the cohort (data not shown). Thus, while the ratio might have some value in risk assessment in cross sectional studies, its use for individual risk assessment is likely to require a more complex approach to algorithm development.

### 4.4. Strengths and Limitations

The strengths of this study included the highly effective weight loss intervention, the menu and recipe defined approach to defining and delivering the low fat and low carbohydrate dietary pattern-based interventions and the monthly collection of anthropometric data and biospecimens. While a non-randomized design is subject to potential confounding, the value of such designs in research on dietary patterns has also been advocated [76].

We report compliance in terms of adherence to the menu and recipe defined dietary patterns that each participant was asked to follow. An alternative approach is to consider compliance in terms of whether the total amount of dietary carbohydrate and fat consumed daily, when adjusted for differences in body size and body composition, was the same among participants within a dietary pattern group. Since total amounts of these macronutrients varied due to the differences that were observed in amount of weight lost among participants, the contribution of total daily amounts of dietary fat and carbohydrate consumed to study results was not determined.

## 5. Conclusions

CHOICE permitted us to look closely at general conclusions from many studies that were either cross sectional or that collected data only at the beginning and end of weight loss. Thus, while leptin is generally stated to be strongly related to fat mass, adiposity fails to explain the majority of the variation in circulating levels of leptin and there are clearly factors unrelated to fat mass *per se* that account for marked changes in plasma leptin during weight loss. CHOICE also revealed unexpected changes in circulating adiponectin and raised questions about how to interpret plasma data relative to the competing factors that determine prognosis in the breast cancer survivor population. Finally, despite many reports about the potential value of the ratio of adiponectin to leptin as an index of effect, we found so much variability within and among individuals who were assessed monthly that we raise a cautionary note about the usefulness of the ratio. The fact that the dietary patterns evaluated failed to alter adipokines during weight loss should not be used to rule out the possibility that dietary pattern effects could be exerted during weight maintenance and/or the possibility that other dietary patterns might exert effects during weight loss/weight maintenance in specific individuals. In designing additional studies, it would be useful to focus on gaps in understanding of the regulation of both adiponectin and leptin gene expression. If weight loss is shown to favorably affect survival prognosis among breast cancer patients, CHOICE findings fail to provide strong support for investigating a causal linkage between adiponectin or leptin and changes in prognosis mediated by weight loss. Mechanisms not directly related to dietary pattern, weight loss, or fat mass appear to play dominant roles in the regulation of circulating levels of these adipokines.

## Supplementary Files

Supplementary File 1
