# Supplementary material for: Impact of Weight Loss on Plasma Leptin and Adiponectin in Overweight-to-Obese Post Menopausal Breast Cancer Survivors
Source: Nutrients. 2015 Jun 26;7(7):5156–76. doi: 10.3390/nu7075156 (PMC4516992; doi:10.3390/nu7075156)
Supplement: Supplementary File 1 [file nutrients-07-05156-s001.docx]

**Supplementary Information**

**Table S1.** Baseline characteristics.

| **Variable** |  | **Control** | **Low Fat** | **Low Carbohydrate** | ***p*-value** |
| --- | --- | --- | --- | --- | --- |
|  |  | ***n* = 53** | ***n* = 73** | ***n* = 66** | **(Global F)** |
| Race | White | 50 (94) | 70 (96) | 72 (94) | 0.82 |
|  | Black | 2 (4) | 1 (1) | 3 (4) |  |
|  | Hispanic | 1 (2) | 1 (1) | 3 (4) | 0.46 |
|  | Other | 1 (2) | 2 (3) | 1 (2) |  |
| Age (years) |  | 57.7 ± 7.6 | 54.5 ± 9.2 | 55.2 ± 8.9 | 0.11 |
| Height (cm) |  | 164 ± 6 | 166 ± 6 | 165 ± 7 | 0.48 |
| BMI (kg/m^2^) |  | 29.2 ± 2.7 | 28.2 ± 2.4 | 29.4 ± 2.5 | 0.01 |
| Weight (kg) |  | 79.7 ± 9.3 | 77.6 ± 7.7 | 79.7 ± 8.6 | 0.24 |
| Fat Wt (kg) |  | 34.9 ± 7.3 | 33.0 ± 5.8 | 35.0 ± 6.0 | 0.11 |
| Fat Mass (%) |  | 43.5 ± 5.3 | 42.4 ± 5.2 | 43.8 ± 4.6 | 0.24 |
| Lean Wt (kg) |  | 44.8 ± 4.8 | 44.6 ± 5.2 | 44.8 ± 5.1 | 0.97 |
| Lean Mass (%) |  | 56.5 ± 5.3 | 57.6 ± 5.1 | 56.3 ± 4.6 | 0.24 |
| Waist (cm) |  | 95 ± 8 | 92 ± 7 | 94 ± 7 | 0.03 |
| Hip (cm) |  | 111 ± 7 | 111 ± 6 | 112 ± 7 | 0.40 |
| RMR (kcal/d) |  | 1297 ± 132 | 1284 ± 136 | 1296 ± 137 | 0.83 |
| Steps (daily) |  | 6257 ± 3027 | 7535 ± 2957 | 7096 ± 2989 | 0.08 |

Values are means ± SD or *n* (%).

**Table S2.** Dietary Composition by Diet Group for Menu Cycle (1200 kcal/day).

|  | **Low Carbohydrate, High Fat** | **High Carbohydrate, Low Fat** |
| --- | --- | --- |
| Calories | 1204 ± 35 | 1186 ± 58 |
| Carbohydrate (g) | 100 ± 4  (32 ± 1) | 193 ± 10  (62 ± 1) |
| Fat (g) | 64 ± 3  (46 ± 1) | 24 ± 2 (17 ±1) |
| Protein (g) | 68 ± 4  (22 ± 1) | 62 ± 6  (20 ± 1) |
| Fiber (g) | 17 ± 4 | 26 ± 5 |
| Sodium (mg) | 2113 ± 741 | 2586 ± 688 |
| Cholesterol (mg) | 244 ± 131 | 120 ± 85 |
| Saturated Fat (g) | 11 ± 2 | 5 ± 2 |
| Monounsaturated Fat (g) | 21 ± 3 | 5 ± 1 |
| Polyunsaturated Fat (g) | 9 ± 2 | 5 ± 1 |
| S:M:P Ratio | 1:2:1 | 1:1:1 |

Values are calories, g or mg ± SD. Values in parentheses are percent energy ± SD. Cycle menus were developed for the following calorie levels: 1200, 1400, 1500, 1600, and 1800 kcal/d. The ratio of n3 to n6 fatty acids was held constant in both intervention diets.

^
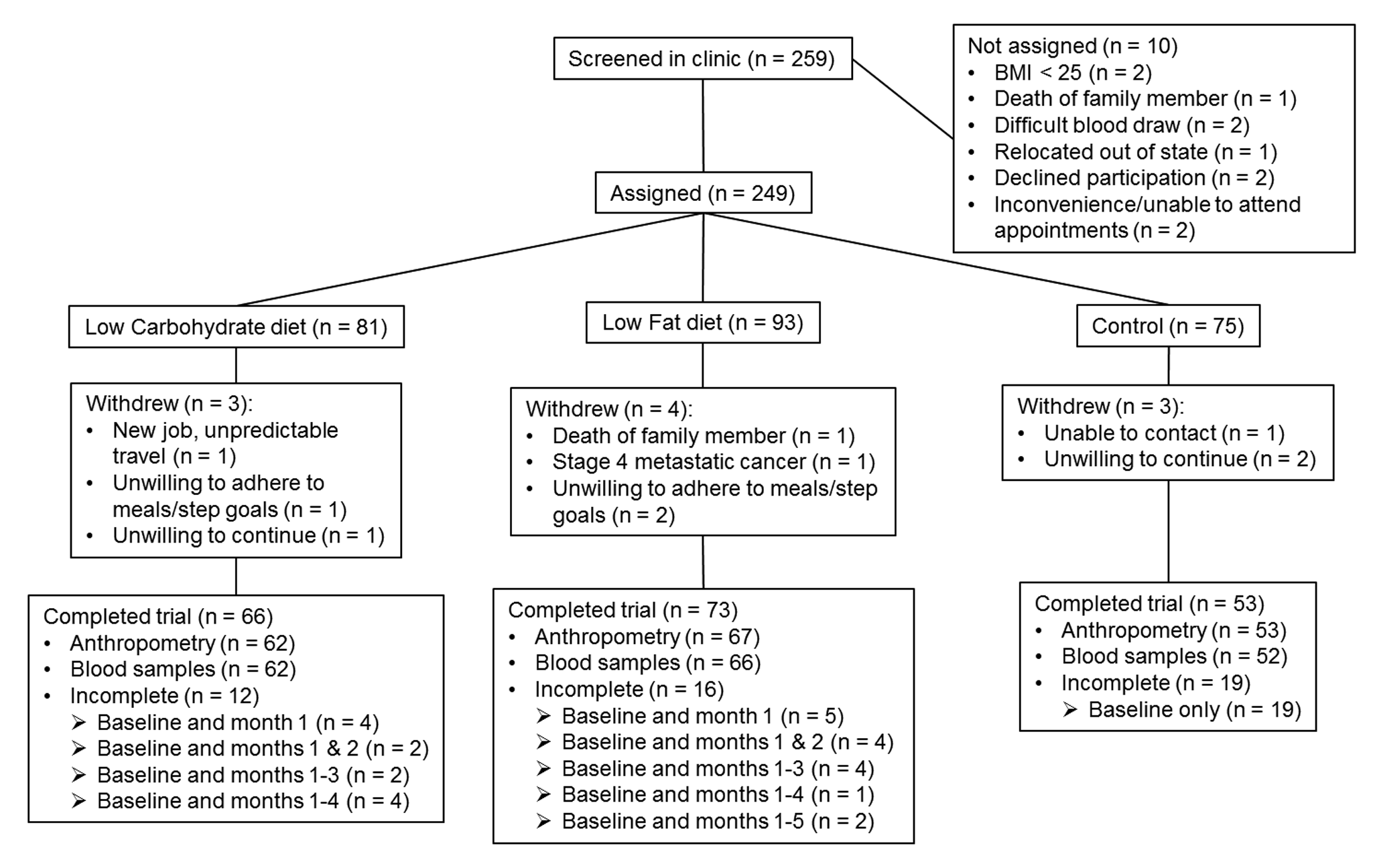
^

**Figure S1.** Flow diagram for screening, assignment, and follow-up of study participants.

| 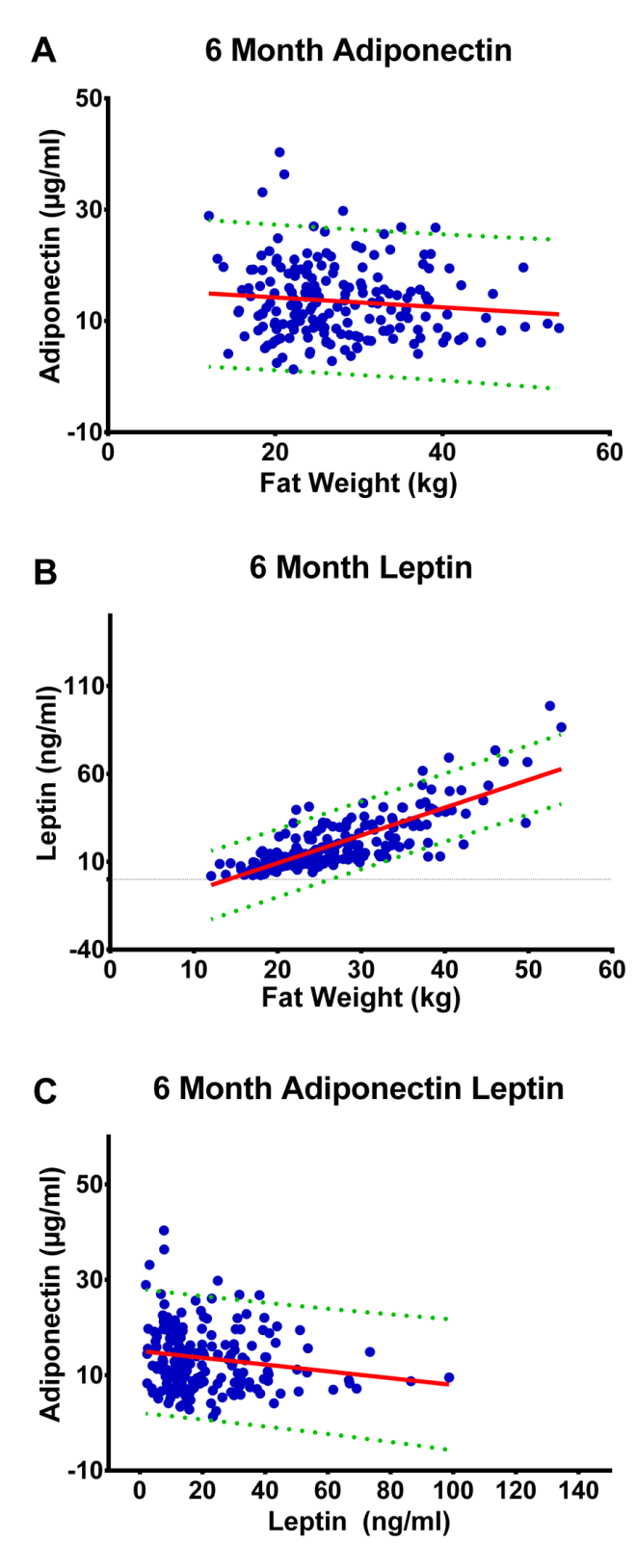 | 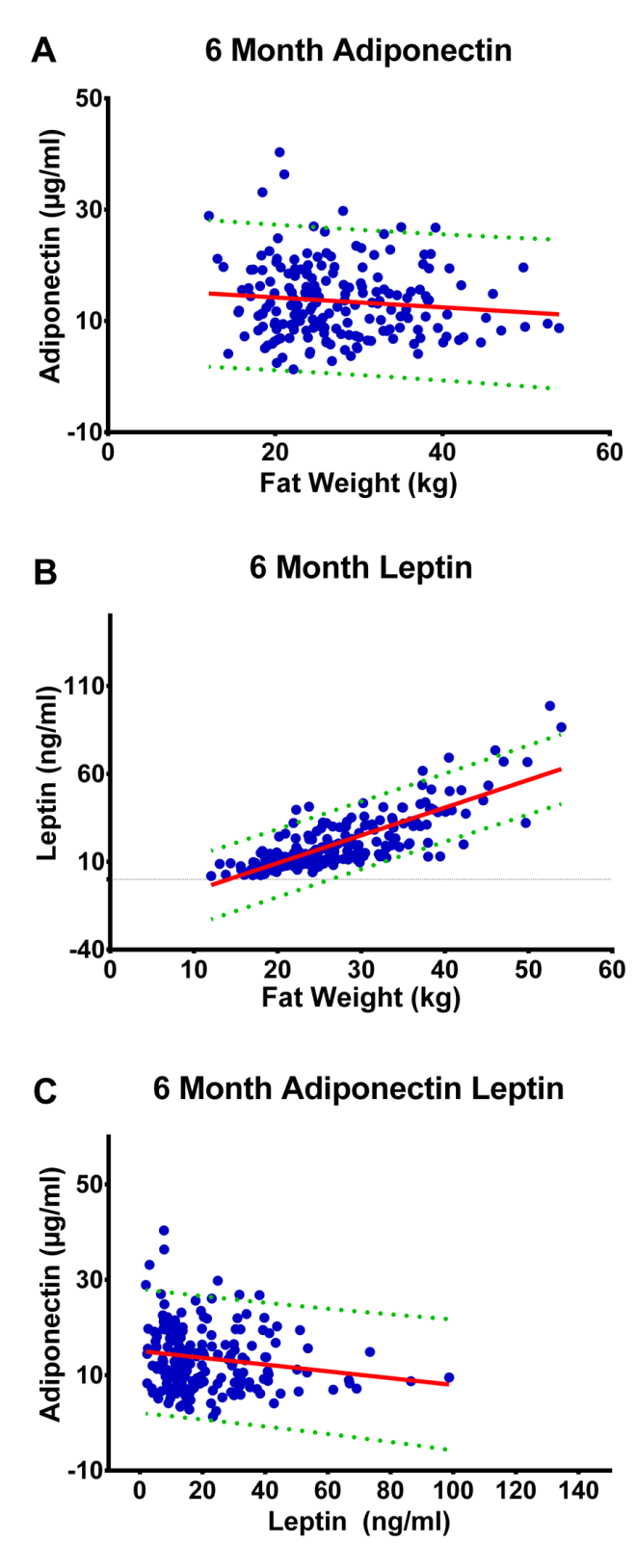 | 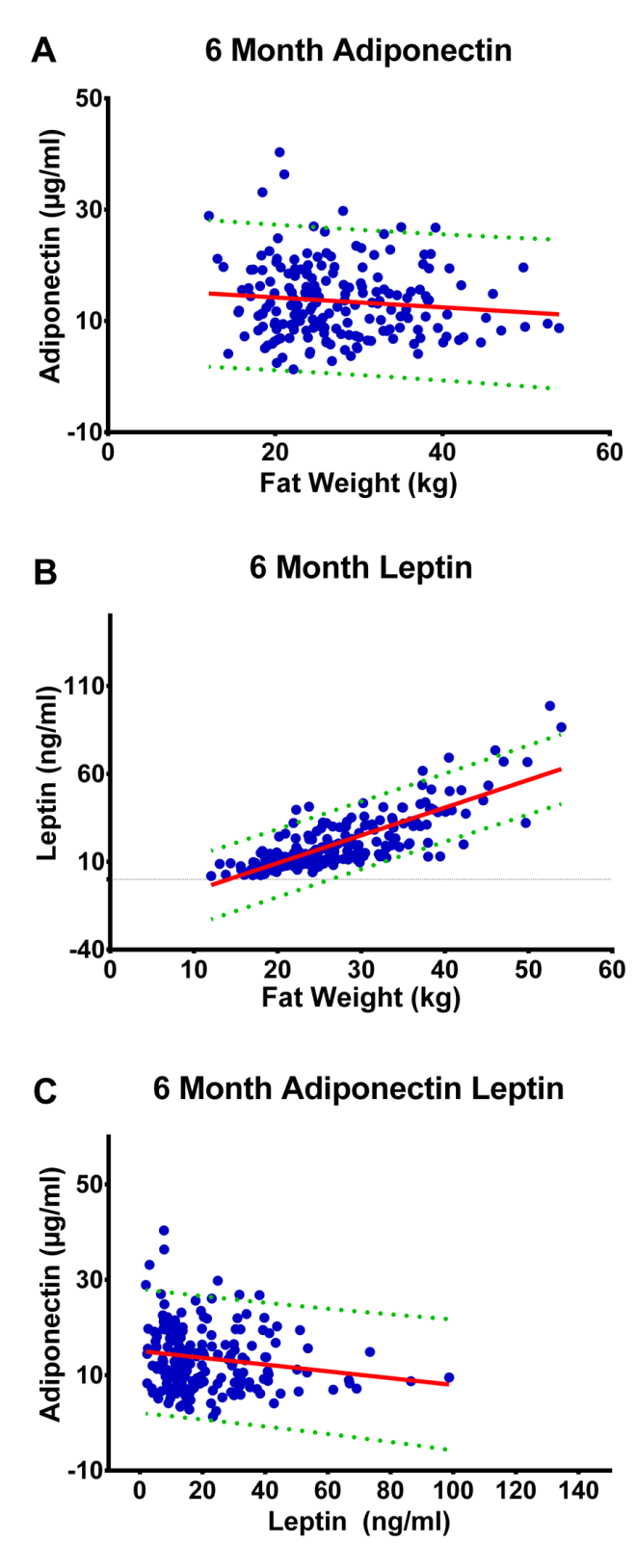 |
| --- | --- | --- |

**Figure S2.** Regression Analyses at end of study. (**a**) Regression of plasma adiponectin on fat mass (kg) with 95% confidence intervals; r^2^ = 0.009, *p* = 0.207. (**b**) Regression of plasma leptin on fat mass (kg) with 95% confidence intervals; r^2^ = 0.631, *p* < 0.001.
(**c**) Regression of plasma adiponectin on plasma leptin with 95% confidence intervals;
r^2^ = 0.024, *p* = 0.032.

© 2015 by the authors; licensee MDPI, Basel, Switzerland. This article is an open access article distributed under the terms and conditions of the Creative Commons Attribution license (http://creativecommons.org/licenses/by/4.0/).
